# Smoking in Pregnancy

**DOI:** 10.1289/ehp.114-a150

**Published:** 2006-03

**Authors:** Hanns Moshammer, Manfred Neuberger

**Affiliations:** Institute of Environmental Health, Medical University of Vienna, Vienna, Austria, E-mail: hanns.moshammer@meduniwien.ac.at

Zanardo et al. (2005) raise an interesting issue in their article, namely, the influence of smoking on immunologic parameters and especially on the composition of human breast milk. The findings deserve notice and further investigation.

Because of the importance of the findings, the errors and inconsistencies in the article (Zanardo et al. 2005) should be corrected. The first inconsistency is related to the number of participants: In the “Materials and Methods,” Zanardo et al. (2005) gave two different numbers:

Of the 1,217 eligible participants, 25 of 26 self-identified as smokers (≥ 5 cigarettes per day through pregnancy until last trimester) were recruited for study participation. One was excluded from the final analysis because of maternal fever. Control participants included consecutive women without history of smoking and matched a smoking participant on the basis of overall inclusion criteria.

We grouped human milk samples into third postpartum day smoker and nonsmoker mother groups, with 42 and 40 samples per group, respectively, and into 10th postpartum day smoker and nonsmoker mother groups, with 42 and 40 samples per group, respectively.

The latter numbers (42 and 40) are also repeated in the first sentence of “Results” and in Table 1. Also in Table 1 the average number of cigarettes smoked per day by the 42 smokers is given as 3.2 ± 0.7 (mean ± SD). This is inconsistent with their selection criterion of “≥ 5 cigarettes per day.”

Table 1 also provides information on birth weight, gestational age, and APGAR score. As expected from numerous previous studies, the children of the smokers scored lower in all these respects. Under “statistical analysis” the authors write that they “used the Student *t*-test for the analysis of [these] data.” To apply the Student *t*-test for the birth weight data seems a sensible choice. When doing this using the figures provided in Table 1, the difference of the birth weight between the children of smokers and nonsmokers is clearly highly significant (*t* = 9.45). In the “Results,” Zanardo et al. (2005) stated erroneously that “the birth weight of the newborn infants of smoker mothers was not significantly lower.”
